# Extended *in vitro* culture of primary human mesenchymal stem cells downregulates *Brca1*‐related genes and impairs DNA double‐strand break recognition

**DOI:** 10.1002/2211-5463.12867

**Published:** 2020-06-09

**Authors:** Xuanwen Bao, Jing Wang, Guangming Zhou, Attila Aszodi, Veronika Schönitzer, Harry Scherthan, Michael J. Atkinson, Michael Rosemann

**Affiliations:** ^1^ Institute of Radiation Biology Helmholtz Center Munich ‐ German Research Center for Environmental Health Neuherberg Germany; ^2^ Medical Graduate School Technical University of Munich Germany; ^3^ State Key Laboratory of Radiation Medicine and Protection School of Radiation Medicine and Protection Medical College of Soochow University Suzhou China; ^4^ Laboratory of Experimental Surgery and Regenerative Medicine Clinic for General, Trauma and Reconstructive Surgery Ludwig‐Maximilians‐University Munich Germany; ^5^ Bundeswehr Institute of Radiobiology Affiliated to the University of Ulm Munich Germany; ^6^ Radiation Biology Technical University of Munich Germany

**Keywords:** *BRCA1*, cellular aging, DNA repair, homologous recombination, mesenchymal stem cells

## Abstract

Mesenchymal stem cells (MSCs) are multilineage adult stem cells with considerable potential for cell‐based regenerative therapies. *In vitro* expansion changes their epigenetic and cellular properties, with a poorly understood impact on DNA damage response (DDR) and genome stability. We report here results of a transcriptome‐based pathway analysis of *in vitro*‐expanded human bone marrow‐derived mesenchymal stem cell (hBM‐MSCs), supplemented with cellular assays focusing on DNA double‐strand break (DSB) repair. Gene pathways affected by *in vitro* aging were mapped using gene ontology, KEGG, and GSEA, and were found to involve DNA repair, homologous recombination (HR), cell cycle control, and chromosomal replication. Assays for the recognition (γ‐H2AX + 53BP1 foci) and repair (pBRCA1 + γ‐H2AX foci) of X‐ray‐induced DNA DSBs in hBM‐MSCs show that over a period of 8 weeks of *in vitro* aging (i.e., about 10 doubling times), cells exhibit a reduced DDR and a higher fraction of residual DNA damage. Furthermore, a distinct subpopulation of cells with impaired DNA DSB recognition was observed. Several genes that participate in DNA repair by HR (e.g., Rad51, Rad54, BRCA1) show a 2.3‐ to fourfold reduction of their mRNA expression by qRT‐PCR. We conclude that the *in vitro* expansion of hMSCs can lead to aging‐related impairment of the recognition and repair of DNA breaks.

## Graphical abstract

During the process of their *in vitro* expansion, primary human BM‐MSCs experience a gradual impairment of their ability to recognize DNA double‐strand breaks. A reduced DNA damage response (fewer γ‐H2AX + 53BP1 foci) was associated with a downregulation of BRCA1‐related DNA repair by homologous recombination and chromosomal replication pathways, suggesting that *in vitro* aged hMSCs could be affected by reduced genetic stability.

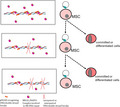

**Abbreviations**
CNV: DNA copy‐number variation
DDR: DNA damage response
DSB: double‐strand break
GO: gene ontology
GSEA: gene set enrichment analysis
hBM‐MSC: human bone marrow‐derived mesenchymal stem cell
HR: homologous recombination
IPA: Ingenuity Pathway Analysis
MSC: mesenchymal stem cell/mesenchymal stromal cell
qRT‐PCR: quantitative real‐time polymerase chain reaction

Mesenchymal stem or mesenchymal stromal cells (MSCs) are adult stem cells that reside in bone marrow stroma and other connective tissues. Their lifelong potential to generate committed precursor cells for various lineages is essential for both the continuous replacement of cellular losses and the recovery of connective tissue damage. In addition to their role as a stem cell pool, MSCs are also a source for immunomodulatory paracrine factors, thereby serving as a regulator of inflammation and immune response [1, 2]. The possibility to expand donor‐ and patient‐derived MSCs *in vitro* and to induce a selected differentiation program prior to an autologous or allogenic implantation makes them highly attractive for cell‐based therapies [3, 4].

The procedure for *in vitro* expansion of MSCs is not yet optimized. Cellular stressors, such as excessive oxygen levels or exposure to low‐dose irradiation, can impair the natural function of MSCs during the subsequent *in vitro* expansions required for transplantation [5, 6]. Sources of genotoxic stress can be as various as repeated diagnostic radiology, therapeutic radiation applications, or long‐lasting low‐level exposures to environmental or occupational noxae, both in the form of ionizing radiation and in the form of chemicals that form DNA damaging radical. One common problem during *in vitro* expansion of MSCs is the appearance of cellular senescence, characterized by a gradual loss of their proliferative capacity and their multipotency [7, 8]. The age and health status of MSC donors also have an influence on the long‐term proliferative capacity of *in vitro ‐*expanded MSCs. The loss of stemness and increased senescence are associated with changes in epigenetic DNA marks [8, 9, 10]. Investigating the detrimental influence of oxidative stress on proliferation and DNA damage response (DDR) of bone marrow‐derived human MSC, Bigot *et al*. (2015) observed that a reduced oxygen environment results in a higher initial clonogenicity, a less‐pronounced loss of colony numbers with increasing passage numbers, and a higher baseline expression of the DNA repair protein RAD51 [11]. They could also show that under reduced O_2_, chemically induced DNA double‐strand breaks (DSB) are repaired more efficiently, with γ‐H2AX foci going back to nearly control level after 24‐h repair incubation. Considering that oxidative stress is indeed a major factor causing cellular aging, one could speculate that their data indeed suggest an age‐related change in DNA repair.

The long residency of MSCs in tissues, and the fact that they cannot be regenerated from more primitive cells, makes MSCs vulnerable to the accumulation of DNA damage by chronic genotoxic stress. Some studies suggest that hMSCs are relatively radioresistant, even after higher radiation doses [12, 13]. MSCs are not prone to radiation‐induced apoptosis, and their robust clonogenicity after ionizing radiation suggests a relatively high radioresistance [14]. A study by Oliver *et al*. [15] found that in comparison with primary osteoblasts, MSCs have a higher DNA repair capacity. This suggests that the radioresistant clonogenicity of MSCs is due to efficient DNA repair mechanisms, rather than resulting from a tolerance of slowly cycling cells to unrepaired DNA breaks. In a previous study, we investigated changes in DNA repair efficiency and genomic stability in murine MSCs during *in vitro* expansion [16]. There was a gradual loss in their ability to recognize both endogenous and radiation‐induced DNA DSB. This impaired DDR was associated with reduced ATM dependency of foci formation, slower DNA repair kinetics, and an increased number of residual DNA repair foci. To gain a more detailed insight into these potentially deleterious age‐related changes, we have conducted a whole‐transcriptome comparison between *in vitro*‐aged and young human BM‐MSCs. The observed dysregulation of key players in DNA DSB repair by homologous recombination (HR) was confirmed using gene expression studies and cell‐based DNA repair assays after X‐irradiation.

## Materials and methods

### Transcriptomic analysis of *in vitro* aging hMSCs

Binary sequence alignment map file of GSE59966 were downloaded from the GEO database [17]. Details of cell origin, *in vitro* aging, RNA extraction, and RNA_Seq can be found in the original paper [17]. SAMTOOLS was applied to obtain FASTQ files [18]. FASTQC was applied to estimate the read quality of the FASTQ file [19]. r software (v 3.5.3, The R Foundation, www.r‐project.org) was used for all the analyses in this study. ‘GenomicAlignments’ package was used to align short reads to a reference genome (human genome hg19). summarizeOverlaps function was applied to obtain the counting matrix. Since the library was prepared by a paired‐end technology, the parameter for alignment was chosen as ‘paired‐end’. Counting model ‘Union’ was chosen as the parameter in the function to indicate those reads that overlap any portion of exactly one feature are counted. ‘DEseq2’ package was applied to perform the differentially expressed gene (DEG) analysis [20]. Gene ontology (GO) analysis and KEGG pathway analysis were done using the cluster Profiler package [21]. The gene set enrichment analysis (GSEA) was done by GSEA software from Broad Institute. Ingenuity Pathway Analysis (IPA; Qiagen, Hilden, Germany) was applied to analyze the canonical pathways.

### Cell isolation and culture

Bone marrow‐derived human MSCs were isolated from femoral heads of healthy donors recruited at the Clinic for General, Trauma and Reconstructive Surgery of the Ludwig‐Maximilians‐University. The study was approved by the LMU ethical commission (project number 238/15) and performed according to the Declaration of Helsinki. All donors signed a written informed consent, declaring that any surplus tissue can be used for medical studies at the university hospital. Cells were isolated by washing the bone graft material with PBS (pH 7.4). Furthermore, the bone material was incubated with 250 U·mL^−1^ collagenase II (Worthington Biochemical Corp., Lakewood, NJ, USA) in DMEM (Life Technologies) for three times for 10 min at 37 °C. To remove bone fragments, both suspensions were filtered with a 100‐µm cell strainer. After centrifugation at 500 ***g*** for 5 min, the cell pellet was resuspended in culture media of αMEM (Thermo Fisher, Waltham, MA, USA) supplemented with 10% FBS (Thermo Fisher Life Technologies, Waltham, MA, USA and 40 IU·mL^−1^ penicillin/streptomycin (Thermo Fisher Life Technologies). Cells were kept growing at 37 °C in a humidified incubator at 5% CO_2_ and ambient oxygen. After 3 days in culture, nonadherent cells were removed by washing with PBS for three times. For *in vitro* expansion and aging of the human bone marrow‐derived mesenchymal stem cell (hBM‐MSCs), cells were passaged every 7 days over a period of 10 weeks in antibiotic‐free growth medium (see above). From week 2 till week 6, cells were split in a 1 : 3 ratio, and from week 7 till week 10 in a 1 : 2 ratio. The entire aging protocol therefore covered eight passages, and from the splitting ratios, it can be estimated to be equivalent to ~ 10.3 cell divisions. Cells of passage 3 are used as ‘young MSCs’ and cells of passage 11 as ‘*in vitro* aging MSCs’, both for mRNA quantification by qRT‐PCR and for immunofluorescence staining. Due to the requirement for a larger cell number, cell cycle analysis and measurement of BrdU incorporation were done on cells after one or two additional passages. Therefore, ‘young MSCs’ refers to cells at passages 3–4 and ‘aging MSCs’ refers to cells at passages 12–13 for these two assays.

### RNA extraction and reverse transcription

Three days after the last passage, the cultured MSCs were washed twice with cold PBS. MSCs were collected using a cell scraper and centrifuged to obtain cell pellets. Maxwell miRNA Kit (Promega, Madison, WI, USA) was used to extract total RNA on a Maxwell 16 AS2000 instrument (Promega, Madison, WI, USA). For reverse transcription, 100 ng of RNA was processed with the SuperScript® III Kit (Thermo Fisher, Waltham, MA, USA) according to the manufacturer’s protocol with random hexamer and oligo‐dT primers.

### qRT‐PCR

qRT‐PCR mix containing the 2 µL cDNA template, 1 µL (5 pmol·µL^−1^) primers (table S1), 10 µL PowerUp SYBR Green Master Mix (Thermo Fisher), and 7 µL nuclease‐free water was pipetted in three technical replicates and run in 96‐well plates on a StepOnePlus instrument (Applied Biosystems, Foster City, CA, USA). The real‐time cycling conditions were adjusted according to the manufacturer’s recommendation. Raw data were collected as.eds files and analyzed using stepone software (v2.3, Applied Biosystems, Foster City, CA, USA) with the ∆∆C*_t_* method and GAPDH as housekeeping gene.

### X‐ray irradiation

X‐ray irradiation was performed in a closed cabinet XStrahl RS225 device (XStrahl Ltd., Surrey, UK) operating at 195 kVp, 1.14 Gy·min^−1^ dose rate, and 3‐mm Al filter. Dose calibration was regularly done using LiF thermoluminescence. Cells were kept at ambient temperature during irradiation, and control cells were treated identically apart from not placing them inside the radiation source.

### Immunofluorescence

The MSCs were plated on sterile glass slides (SuperFrost+; Carl Roth GmbH, Karlsruhe, Germany) 2 days before X‐irradiation at 30% cell density. At the indicated time points after irradiation, medium was removed and the slides rinsed with cold PBS. After fixing the cells for 10 min at ambient temperature in Roti‐Histofix 4.5 (Carl Roth GmbH, Karlsruhe, Germany), slides were incubated for 1 h in PBS Plus (BSA 1 g, glycine 0.5 g, PBS 100 mL) to block the unspecific binding of first antibody. Incubation with the first antibody (53BP1: NB100‐305, Novus Biologicals, Littleton, CO, USA; pBRCA1: ab90528, Abcam plc., Cambridge, UK; γ‐H2AX: 05‐636, Merck Millipore, Burlington, MA, USA) diluted in PBS Plus (dilution ratio: 53BP1: 1 : 200, pBRCA1: 1 : 200, and γ‐H2AX: 1 : 400) was done in a humidified chamber overnight at 4 °C. The slides were then washed 3 x 1 5min in PBS, before incubating them with 150 µL of secondary antibody solution. Secondary antibody solution was prepared by mixing Alexa Fluor® 488‐conjugated goat anti‐rabbit IgG H&L or Cy3‐conjugated goat anti‐mouse IgG H&L antibody (both from the Jackson ImmunoResearch, West Grove, PA, USA) in PBS Plus in a 1 : 400 dilution. After 1‐h incubation at room temperature in the dark, the slides were washed again 3 x 15 min in PBS. For counterstaining of nuclei, the slides were covered with 150 µL of a DAPI solution (0.3 µm in PBS). After 10‐min incubation at RT, the DAPI solution was removed, and slides were rinsed 2x with PBS and covered with Vectashield (Vector Labs, Burlingame, CA, USA) and glass coverslips. Fluorescence microscopy was done on a digital BZ‐9000 microscope (Keyence, Itasca, IL, USA). Foci were counted automatically using the Keyence BZ‐II analyzer. For each experiment, at least 50 cells were counted.

### Analysis of cell cycle distribution and kinetics

For analysis of changes in the cell cycle distribution, MSCs at day 2 after plating were trypsinized, washed with 4ml ice‐cold PBS, and spun down at 300 ***g***  for 5 min. Following removal of the supernatant, cells were resuspended in 4 mL of ice‐cold 70% EtOH for 3 h. The fixed cells were spun down at 300***g*** for 5 min and resuspended in 475 μL PBS, plus 3.3 μL RNAse A (30 mg·mL^−1^) and 25 μL propidium iodide (1 mg·mL^−1^, both from Sigma‐Aldrich, Taufkirchen, Germany). Flow cytometry was done using a Becton Dickinson LSRII (Franklin Lakes, NJ, USA), with 531 nm green‐yellow laser excitation and 610 nm/20 nm band‐pass filter for the PI fluorescence emission. Signals from 5 × 10^5^ cells were stored from the main forward and side scatter population, and cells of G1 and G2/M‐phase were counted using Gaussian curve fitting for the 2*n* and 4*n* peak, whereas S‐phase and apoptotic cells were quantified by subtracting G1 and G2/M from the original histogram.

For quantification of DNA synthesizing cells and S‐phase kinetics, 10^4^ MSCs were plated on sterile glass slides and incubated for 2 days in growth medium. BrdU (Sigma‐Aldrich GmbH,Munich, Germany) was added to a final concentration 10 μg·mL^−1^, and cells were allowed to incorporate the nucleotide analog for 0, 24, and 48 h under standard growth conditions. After the indicated incubation times, the slides were washed twice with PBS before staining for 15 min with Hoechst 33342 solution (20 mm final concentration; Sigma‐Aldrich GmbH). Using UV fluorescence microscopy (see above), 200 cells from randomly selected areas of each slide were photographed and the number of bright versus dim stained nuclei was counted.

## Results

### Bioinformatic analysis of young and aging human MSCs

**Fig. 1:**
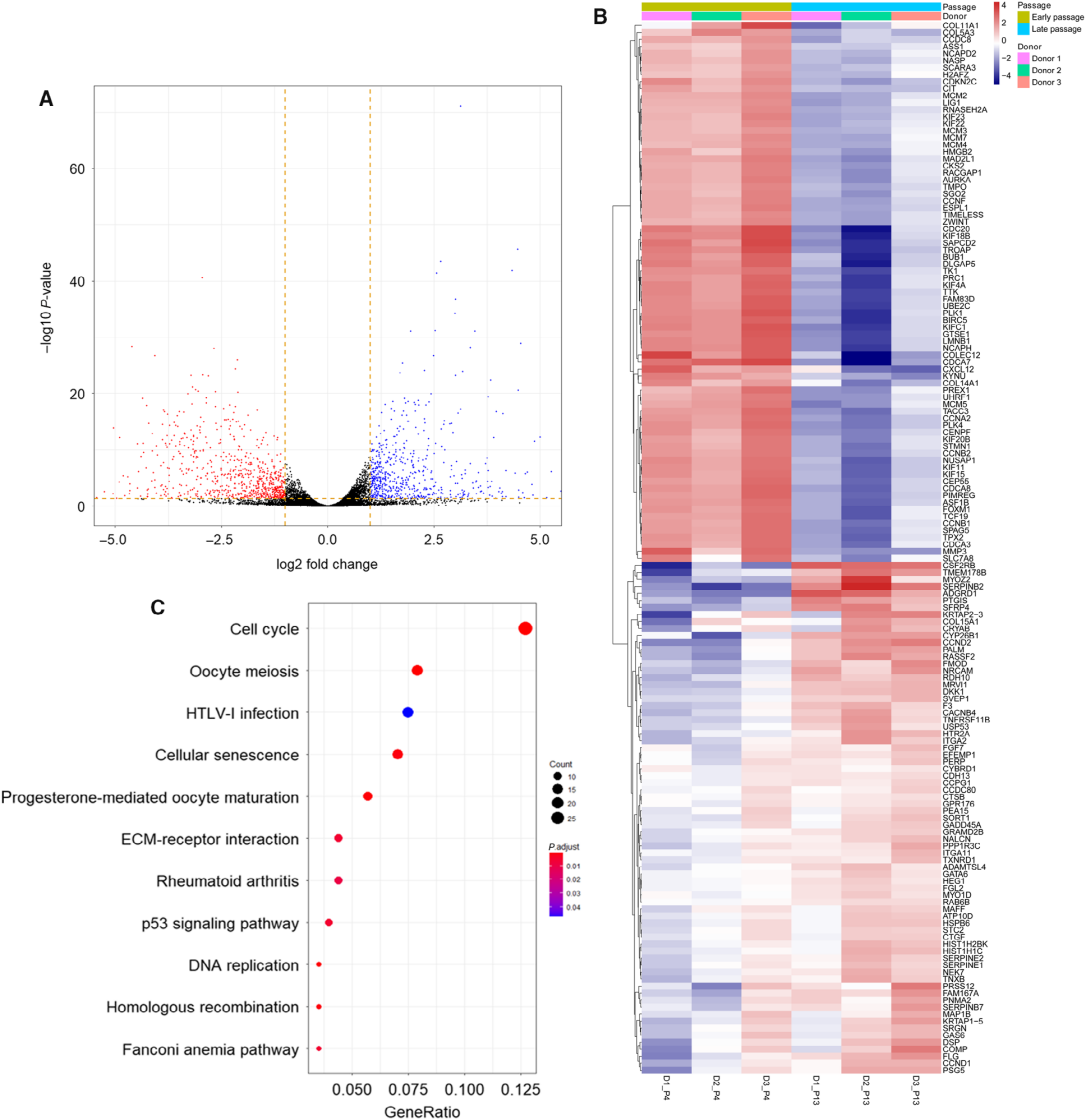
DEG analysis for young and *in vitro* aging hMSCs. (A) Volcano plot showing DEGs between young hMSCs and *in vitro* aging hMSCs from GSE59966. (B) Heat map showing the most significant genes from GSE59966. (C) KEGG plot based on DEGs.

**Fig. 2:**
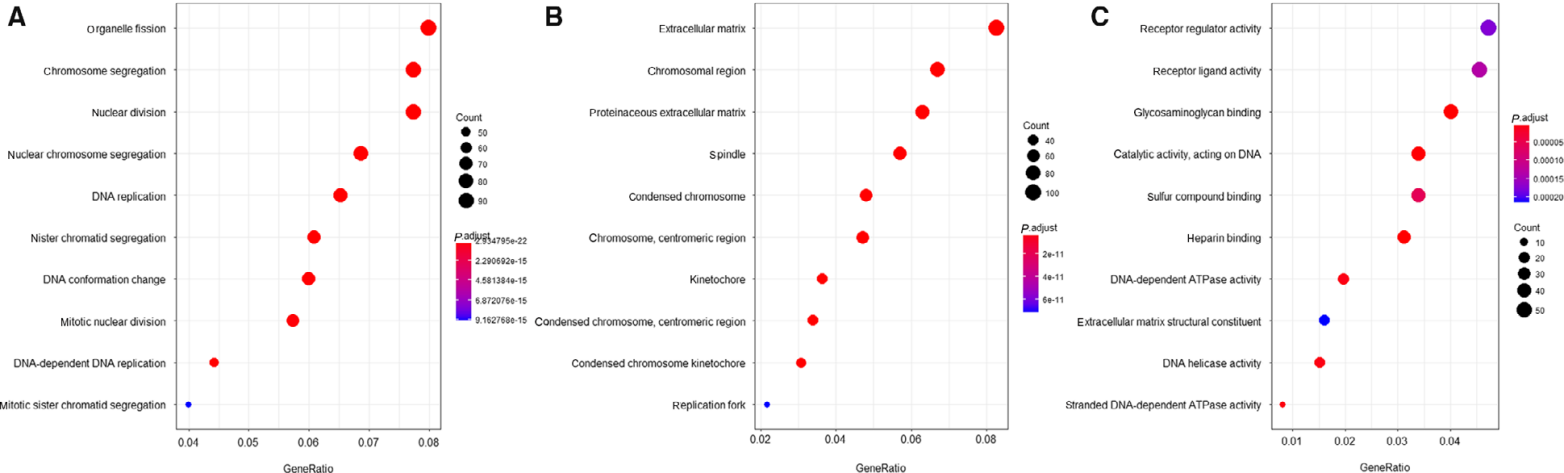
GO analysis for young and *in vitro* aging MSCs from GSE59966. (A) GO analysis for biological processes. (B) GO analysis for cellular components. (C) GO analysis for molecular functions.

RNA‐seq was performed to filter out differentially expressed genes (DEGs). The quality of the sequencing was confirmed by FASTQC (data available on request). PCA plots of the dysregulated genes during *in vitro* aging of six hMSC samples showed that the individual samples clustered together before *in vitro* aging and formed a second cluster after aging (Fig. S1). A volcano plot of the mean values of the relative gene expression changes revealed that the aging process induced changes in the expression of 1308 genes (Fig. 1A) (threshold set for an absolute Log_2_ fold change larger than 1 and a *Q*‐value < 0.05). The heat map of the age‐related DEGs (Fig. 1B) displays the top 150 genes with the highest significance ranked according to their DEG values. Unsupervised hierarchal clustering of the DEGs derived from cells of different donors confirmed the homogeneity in young and aging hMSCs, respectively. KEGG pathway analysis of genes with expression changes above the threshold shows that cell cycle regulation, cell senescence, and DNA repair‐related pathways are the most important dysregulated pathways during aging (Fig. 1C and Data S1: gene list). GO analysis of biological process, molecular function, and cellular process of 1308 significantly dysregulated genes returned enriched sets of molecular functions to cellular processes of organelle fission, chromosome segregation, nuclear division, and other chromosome‐related processes during *in vitro* aging (Fig. 2A‐C). Taken together, the GO and KEGG analyses suggest that apart from already‐known pathways involved in stem cell aging, changes in DNA repair and chromosome construction pathways are also affected by the *in vitro* aging process of hMSCs.

**Fig. 3:**
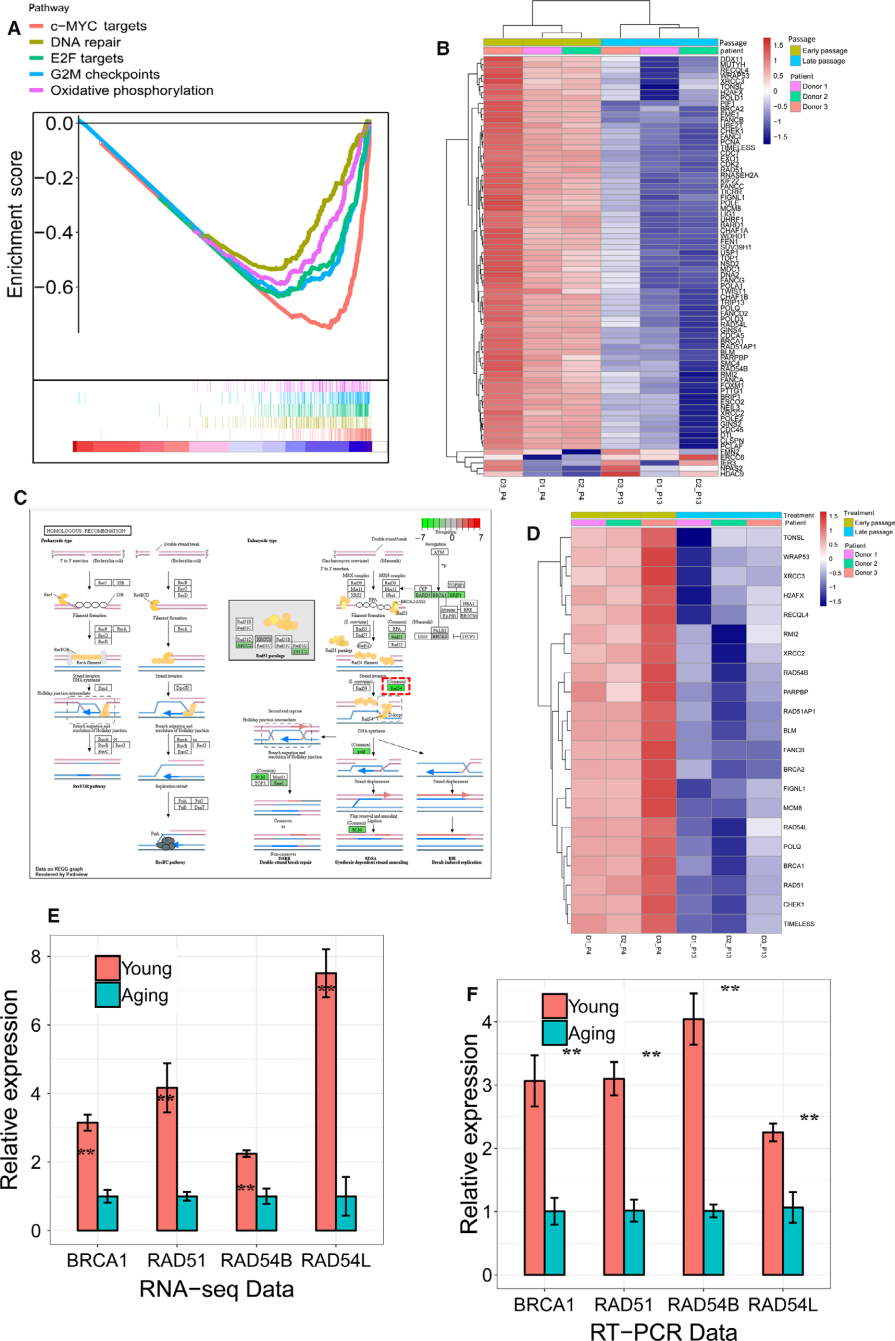
Decreased expression of HR ‐related genes in *in vitro* aging hMSCs. (A) GSEA showing DNA repair and other enriched hallmarks between young and *in vitro* aging hMSCs from GSE59966. (B) The heat map for the significant DNA repair‐related genes between young and *in vitro* aging hMSCs. (C) The decreased gene expression in *in vitro* aging hMSCs in RNA‐seq showing by KEGG plot. (D) The most significantly changed HR‐related genes between young and *in vitro* aging hMSCs by RNA‐seq. The expression‐level change in BRCA1, RAD51, RAD54L, and RAD54B between young and *in vitro* aging hMSCs by RNA‐seq (from GSE59966) (E) and RT‐PCR (F). The expression of target genes in aging MSCs was set arbitrary to 1 (mean values ± SEM, *n* = 3, **P < 0.01, significance by paired, one‐sided *t*‐test).

Gene set enrichment analysis identified DNA repair, G2M checkpoints, E2F target, c‐MYC targets, and oxidative phosphorylation as being influenced by *in vitro* aging (Fig. 3A). A heat map of the relative changes in DNA repair‐related gene expression (Fig. 3B) reveals that most genes involved in DNA repair processes were downregulated.

### Downregulation of genes involved in homologous recombination repair

In GSEA, we found DNA repair to be dysregulated during aging (Fig 3A) and KEGG analysis suggested an effect on HR (Fig. 1C). The components involved in HR repair were examined, and several key players (*RAD51*, *RAD54B, RAD54L,* and *BRCA1*) were found to be strongly downregulated (Fig. 3C‐E). qRT‐PCR in hBM‐MSCs confirmed a downregulation between 2.3‐fold (RAD54L) and 4‐fold (RAD54B) of these genes during their *in vitro* expansion between week 2 and 10 (Fig. 3F). String network analysis placed *RAD51* and *BRCA1* as central nodes of HR (Fig S2A). IPA canonical pathway analysis of the DEGs revealed that cellular processes of ‘cell cycle control of chromosomal replication’ and ‘BRCA1‐mediated DDR’ are the most significantly affected signaling pathways during *in vitro* aging of hMSCs (Fig. S2B).

### Cell cycle distribution and kinetics of actively replicating cells

Flow cytometry analysis of cell cycle distribution in early versus late passage hMSCs indicates no systematic changes related to the duration of *in vitro* aging (Fig. S3A). Of the three donors analyzed, the percentage of S + G2/M cells, which might influence the portion of cells using HR repair, was 23.92%, 28.84%, and 22.18% in the early passages, and 27.57%, 24.80%, and 23.34% in the late passages, respectively (Fig. S3B). Analysis of active DNA synthesis in S‐phase using BrdU incorporation and Hoechst 33342 quenching shows that, within 24 h, (20.8 ± 2.1)% of early passage MSCs and (17.8 ± 1.5)% of late passage MSCs complete S‐phase. During 48 h, the percentage of DNA‐synthesizing cells increased to (37.1 ± 2.2)% or (35.6 ± 4.8)% in the early and late passages, respectively (difference not significant) (Fig. S4A,B). Although there appears to be a fraction of > 50% slowly or not cycling cells, the comparison shows that this is unrelated to the passage number *in vitro*.

### Impaired DNA damage response during hMSC aging *in vitro*

**Fig. 4:**
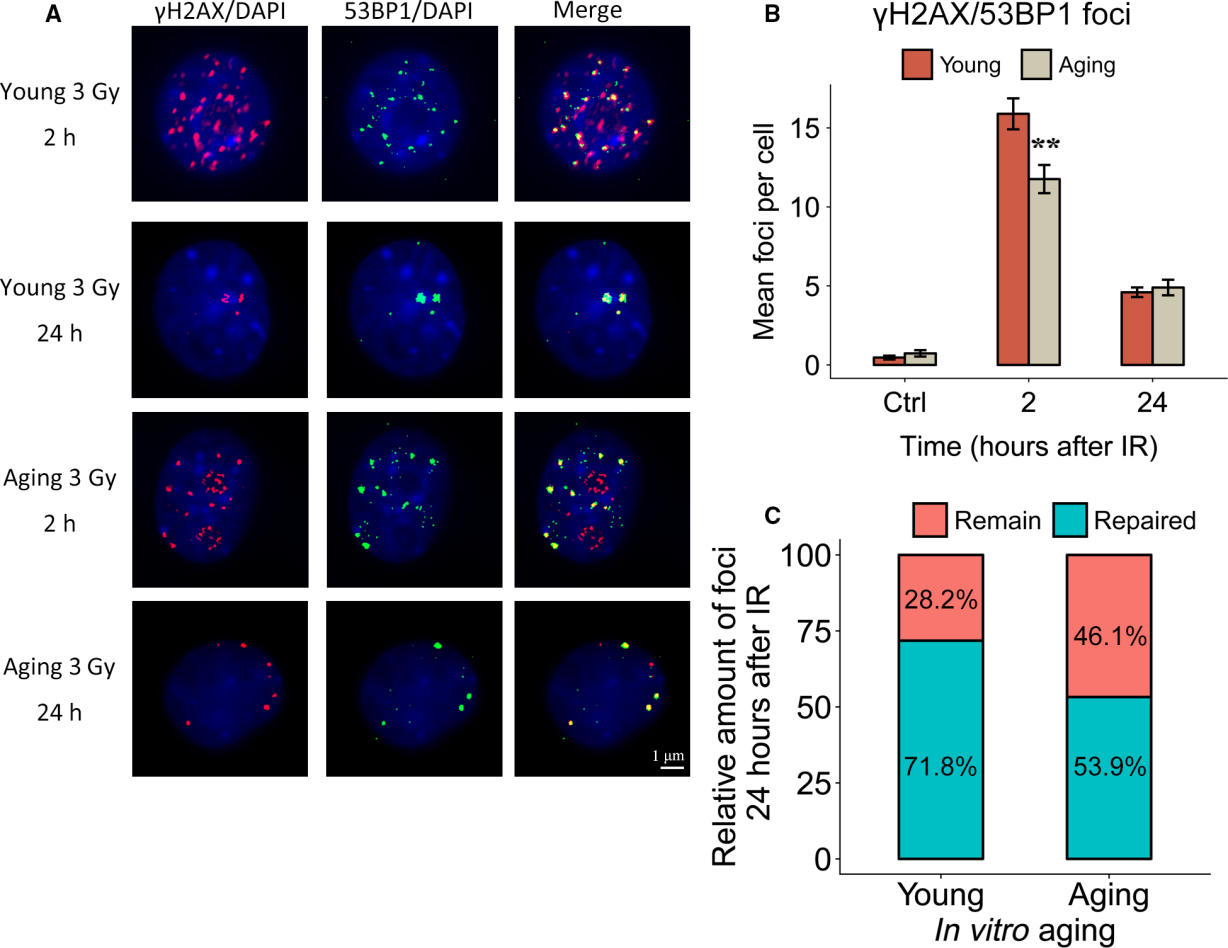
Impaired DNA repair capacity in *in vitro* aging hMSCs. (A) DSB damage foci (γH2AX, red; 53BP1, green) formation shown in young and *in vitro* aging MSCs in control, 2 and 24 h after 3 Gy of γ‐irradiation groups. Nuclear counterstaining by DAPI. Length of the scale bar: 1 µm. (B) Quantification of γH2AX + 53BP1 DSB foci in MSCs 2 and 24 h after γ‐irradiation (mean values ± SEM, *n* = 3, ** *P* < 0.01, significance by paired, one‐sided t‐test). (C) Percentage of colocalized γH2AX + 53BP1 foci 24 h postirradiation relative to the values at 2 h postirradiation in young and *in vitro* aging MSCs.

To test whether the observed reduction in the expression of genes involved in HR has a functional consequence for DNA repair in *ex vitro*‐expanded MSCs, we stained X‐irradiated hBM‐MSCs from different passage numbers for γ‐H2AX/53BP1 DSB repair foci (Fig. 4A). The mean number of γ‐H2AX/53BP1 foci 2 h after 3 Gy irradiation was 16.5 (CI 15.8–17.2) in young and 12.3 (CI 11.4–13.2) in aged cells (*P* < 0.01). Sham‐irradiated cells of the same passage did not exhibit a significant difference in the background level of γ‐H2AX/53BP1 foci (Fig. 4B). A higher proportion of unrepaired foci were seen to be retained in *in vitro*‐aged hBM‐MSCs (46.1%) compared to young cells (28.2%) 24 h after irradiation (Fig. 4C). These results confirmed the impairment of DNA damage sensing and repair during hMSC aging.

### Changes in DNA repair foci involved in homologous recombination

**Fig. 5:**
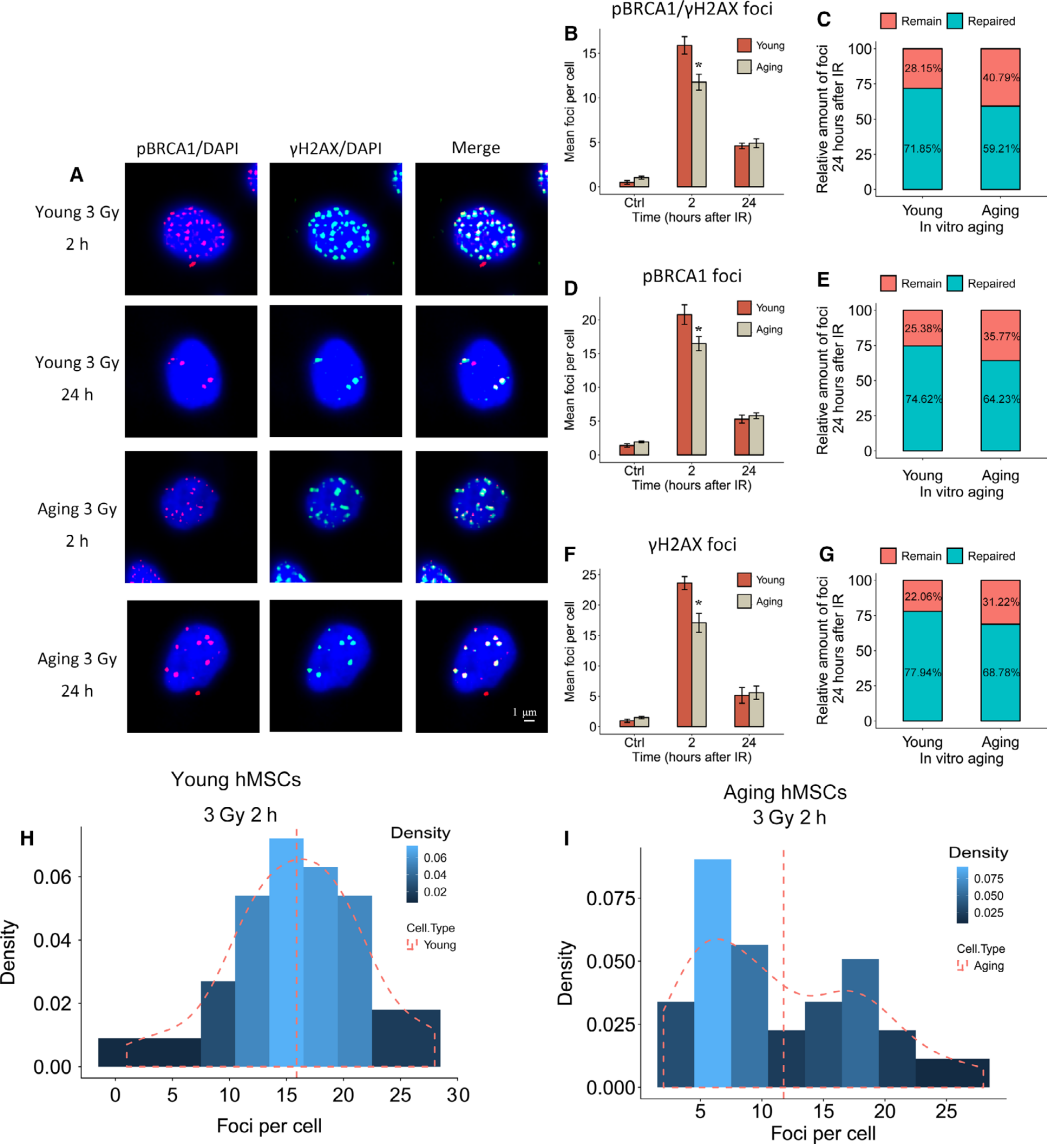
Impaired HR repair capacity in *in vitro* aging hMSCs. (A) Repair foci formation is shown in young and *in vitro* aging hMSCs 2 and 24 h after 3 Gy of γ‐irradiation by immunofluorescence staining for pBRCA1 (red) and γH2AX (green). Nuclear counterstaining by DAPI. Length of the scale bar: 1µm. (B) Quantification of pBRCA1 + γH2AX foci formation in hMSCs 2 and 24 h after γ‐irradiation. (C) The proportion of individual pBRCA1 + γH2AX colocalizing foci in young and *in vitro* aging MSCs 24 h after γ‐irradiation. (D) Quantification of pBRCA1 foci formation in hMSCs 2 and 24 h after γ‐irradiation. (E) The proportion of pBRCA1 foci in young and *in vitro* aging MSCs 24 h after γ‐irradiation. (F) Quantification of γH2AX foci in hMSCs 2 and 24 h after γ‐irradiation. (G) The proportion of individual γH2AX foci in young and *in vitro* aging MSCs 24 h after γ‐irradiation. (H) Dispersion analysis of DNA repair foci in single cell from young hMSCs. (I) Dispersion analysis of DNA repair foci in single cells from *in vitro* aging hMSCs (mean values ± SEM, *n* = 3, *P < 0.05, significance by paired, one‐sided *t*‐test).

Radiation‐induced DNA DSBs can be repaired by different pathways that are initiated by recognition of the damaged site and the rapid formation of γ‐H2AX/53BP1 foci. As MSCs *in vitro* are proliferating, they preferentially use HR to repair DNA DSBs. To measure DNA repair via HR, the formation of pBRCA1 foci (alone or in colocalization with γ‐H2AX) was compared in young and in *in vitro*‐aged hBM‐MSCs (Fig. 5A). This confirmed that 2 h after irradiation, the older hBM‐MSCs exhibited a lower number of pBRCA1 + γ‐H2AX foci compared to younger hMSCs (Fig. 5B). A similar reduction with *in vitro* age was also seen for pBRCA1 and γ‐H2AX foci counted separately (Fig. 5D‐G). The effective repair of DNA DSBs can be inferred by the subsequent disappearance of the DNA repair foci over a period of 24 h. It was evident that a larger portion of DNA DSBs remained unrepaired in *in vitro* aged hBM‐MSCs as compared to their early passage counterparts (Fig. 5C,E,G). We also analyzed the distribution of the initial DNA repair foci in single cells. After 9 weeks in culture, 16.5% of the MSCs belonged to a clearly separated subset of individual cells with reduced foci number (Fig. 5H‐I). These findings suggest that during *ex vivo* expansion and aging of hBM‐MSCs, a subpopulation arises with a reduced DDR.

## Discussion

Cell‐based therapies using mesenchymal stem cells require *in vitro* expansion prior to transplantation [22, 23]. The cellular aging that accompanies *in vitro* proliferation reduces the capacity of the MSCs both for colony formation and for lineage‐specific differentiation [24]. However, there may also be a deleterious influence of *in vitro* aging on the capacity for genomic maintenance, essential for preventing an accumulation of cytogenetic damage. After successful transplantation, the engrafted MSCs will be subject to genotoxic stress, for example, from ionizing radiation in the form of natural low‐dose background irradiation or from medical exposures. We have recently estimated that such an exposures can accumulate several hundreds of mGy over the life span of an adult stem cell [25]. A study by Ulyanenko *et al*. (2019) provided evidence that gamma‐irradiation of hBM‐MSCs using a low dose‐rate exposure (0.1 mGy·min^−1^) was not sufficient to trigger a DDR, whereas irradiation with the same total dose (300 mGy) at an acute high dose‐rate scheme (30 mGy·min^−1^) strongly induced a cellular response [26].

In our previous study on DDR in murine MSCs, we found that the initial ATM‐mediated recognition of DNA DSBs formed after an exposure to ionizing irradiation was impaired after long‐term *ex vivo* cell expansion [16]. This impaired DDR, indicated by a reduced number of initial γ‐H2AX/53BP1 DSB repair foci, was associated with slower DNA repair kinetics and with an increase in the frequency of radiation‐induced micronuclei. Consistent with the idea that impaired DNA repair increases the long‐term risk for gene mutations leading to tumorigenesis [27], it has recently been found that cells derived from *ex vivo* ‐expanded rodent MSCs can undergo malignant transformation more frequently. MSCs of human origin reportedly are more resistant to transformation [28, 29]. It would therefore be interesting to determine whether the resistance of human MSCs for *in vitro* transformation could be related to their DNA repair potential, and if so, how this is influenced by extended *in vitro* aging. Few studies have addressed DDR and DNA repair in human MSCs. Bigot *et al*. (2015) found in their study that hypoxia (2% oxygen) during *in vitro* culture caused an increased expression of proteins involved in HR repair (*BRCA1*, *RAD51,* and *RBBP8*) and an upregulation of HR activity in hBM‐MSCs. Although hypoxia caused a reduced p53 activation at early passages in BM‐MSCs following doxorubicin treatment, efficient recognition of DNA damage was observed. Doxorubicin is a DSB‐inducing radiomimetic agent and is widely used as a cytotoxic drug in cancer treatment [11, 30], but its retention in cells is also influenced by the expression of membrane efflux pumps such as ABCG2. The high expression of ABCG2 in normal and malignant stem cells is an established and widely used marker leading to the excretion of DNA dyes (Hoechst 33342 side population) and export of cytotoxic molecules, thus reducing the intracellular concentration of such drugs [31, 32, 33]. Their experimental approach might therefore be confounded in part by changes in the stem cell‐specific expression of detoxifying efflux pumps ABCG2, which is known to actively purge cells of doxorubicin. Ionizing radiation, in contrast, is a more homogeneous inducer of DNA lesions, thus avoiding the bias of nonuniform drug concentrations in different cell types.

Therefore, we focused here on the dysregulation of DNA repair pathways in human MSCs after X‐irradiation. Starting from a re‐analysis of transcriptome changes during *in vitro* aging of hBM‐MSCs using various bioinformatics tools, we observed that gene networks involved in DNA repair by HR were impaired during the *in vitro* aging of human MSCs. Chromosome reconstruction and dysregulation of genes in DNA repair pathways were indeed associated with a reduction of DDR in hMSCs. A lower number of γ‐H2AX/53BP1 DSB repair foci may indicate a reduced initial DDR in aged hBM‐MSCs relative to young cells. We have discussed before that this is more likely an indicator of an impaired DDR rather than reflecting a lower number of breaks on the same DNA target level [16]. This was also in line with the observations that aged MSCs with lower number of initial γ‐H2AX/53BP1 DSB repair foci developed more micronuclei after cell division.

Among several DNA repair pathways, HR involving BRCA1 has been identified here as the major process affected during *in vitro* aging of hBM‐MSCs. This is also consistent with results from IPA, which ranked ‘cell cycle control of chromosomal replication’ and ‘BRCA1‐mediated DNA damage control’ as two main processes affected by *in vitro* aging. Since DNA repair by HR is specific for cells in the S‐ and G2 cell cycle phase, an influence of cell cycle control would be a natural consequence. We did not observe, however, significant differences related to the passage number, neither in the cell cycle distribution of unirradiated hBM‐MSCs nor in the percentage or kinetics of actively replicating cells. This might in part be the result of the short time between plating the cells and doing the various assays, thereby avoiding the accumulation of senescent cells. Another possible factor to be considered would be age‐related differences in radiation‐induced cell cycle arrest. Our observation, however, that the difference between young and aged cells in the number of their radiation‐induced 53BP1/ γ‐H2AX and pBRCA1 foci was most pronounced at 2 h after irradiation makes this idea unlikely.

Alterations in the way MSCs regulate the cell cycle most likely happen during the evolution of genetically unstable clones. Using a combination of clonal expansion of human MSCs with cytogenetic, DNA copy‐number variation (CNV), and transcriptome analyses, Wang et al (2014) observed a clone with aneuploidy and large number of CNVs displayed significant downregulation of cell cycle control, BRCA1‐related DDR, and ATM signaling, whereas in a clone without genetic alterations, the same pathways were upregulated [29]. The clonal heterogeneity of age‐related dysregulation found in their study might underlie the formation of a subset of *in vitro*‐aged hBM‐MSCs, which in the present report display a reduced DDR signal after X‐irradiation.

DNA repair foci consisting of colocalized pBRCA1 and γ‐H2AX protein foci specifically indicate sites of HR repair, and those foci appeared here with reduced frequency in X‐irradiated aging hBM‐MSCs as compared to irradiated young hBM‐MSCs. It can be excluded that this is caused by a faster repair in aged cells, since they display a relatively higher fraction of unrepaired DNA DSBs after 24 h. In previous studies, HR has been linked to the radioresistant phenotype of MSCs [12, 34]. Another study found that MSCs deficient in the Fanconi anemia protein FANCD2, which forms a complex during HR with BRCA1 and BRCA2, exhibited a higher level of DNA fragmentation between 1 and 24 h after Cs137 gamma‐irradiation, highlighting the importance of homology‐directed DSB repair specifically for those cells [35]. In our analysis, we observed that both HR and the Fanconi anemia pathway were affected during *in vitro* aging. The higher fraction of residual of pBRCA1 + γ‐H2AX foci in *in vitro* ‐expanded versus young hBM‐MSCs suggests that *in vitro* aging had a detrimental impact not only on the recognition of DNA DSBs, but also on the efficiency of their repair by HR. We also saw that in contrast to early passage hBM‐MSCs, their aged counterparts show a pronounced nonuniform pBRCA1 + γ‐H2AX focus response after X‐irradiation, with more than 50% of cells forming a distinct subset with less initial foci than expected from a random distribution. To find out whether those with atypically low number of initial foci are related to cells showing less complete repair 24 h later, a time‐resolved study on individual cells would be required. But also without such a technically challenging method, we can conclude that both the initial recognition of DNA breaks (via ATM signaling) and efficiency of HR are important for DNA repair in hBM‐MSCs and that their *in vitro* aging can cause a reduction in both of them.

## Conclusions

We confirmed that human MSCs from healthy donors also undergo a gradual reduction of DDR during extended *in vitro* growth. Of the several genes that were predicted by mRNA‐seq and pathway analysis involved in DNA repair by HR, *BRCA1*, *Rad54,* and *Rad51* were experimentally confirmed to show a significant downregulation. Analysis of pBRCA1 + γH2AX foci after X‐irradiation revealed that with increasing duration of *ex vivo* growth, a subpopulation of cells develop that have a distinct lower capacity of damage recognition. It can therefore be concluded that human MSCs *in vitro* show a similar age‐related impairment of the DDR as found previously in murine cells, and that the higher resistance hMSC against malignant transformation *in vitro* must be due to other cellular processes.

## Conflict of interest

The authors declare no conflict of interest.

## Author contributions

MJA and MR conceptualized the study. MR and HS contributed to methodology. XB contributed to software. JW performed validation. XB performed formal analysis. MR investigated the study. AA and VS collected resources. MR and XB performed data curation. XB wrote the original draft. HS, AA, VS, and MR wrote, reviewed, and edited the manuscript. XB performed visualization. MJA and MR underwent supervision. MR contributed to project administration. XB performed funding acquisition.

## Supporting information


**Fig. S1.** PCA analysis showing the distribution of hMSC samples by their RNA transcriptome data.

**Fig. S2.** IPA analysis and gene network for the changed gene in *in vitro* aging process in hMSCs.

**Fig. S3.** Cell cycle analysis of P3/P4 and P12/P13 MSCs using propidium iodide staining and flow cytometry.

**Fig. S4.** BrdU incorporation for 24 and 48 h.

**Table S1.** List and nucleotide sequence of primers used for qRT‐PCR. List and nucleotide sequence of primers used for qRT‐PCR.

**Data S1.** List of the differentially expressed genes from dataset GSE59966 by their log_2_‐FC values.

## Data Availability

All primary data are available upon request. The result of the transcriptome analysis (List of genes with significant fold change) has been uploaded to the journal website (Supplementary_log_2_foldchange_1.csv).
