# Supplementary figures and images for: Extended in vitro culture of primary human mesenchymal stem cells downregulates Brca1‐related genes and impairs DNA double‐strand break recognition
Source: FEBS Open Bio. 2020 Jun 9;10(7):1238–50. doi: 10.1002/2211-5463.12867 (PMC7327915; doi:10.1002/2211-5463.12867)

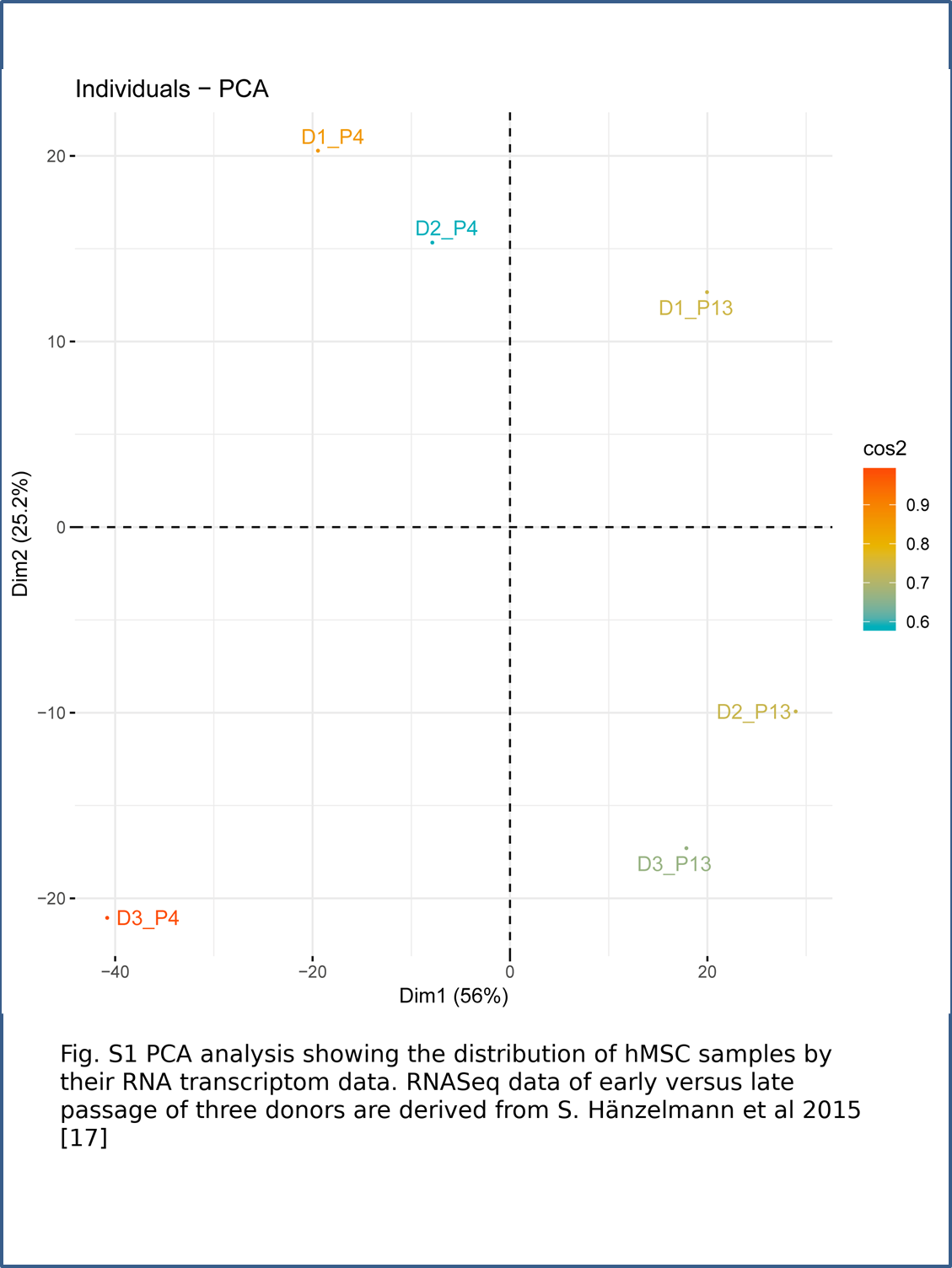

Supplement: Supplementary file 1 — Fig. S1. PCA analysis showing the distribution of hMSC samples by their RNA transcriptome data. [file FEB4-10-1238-s001.tif]

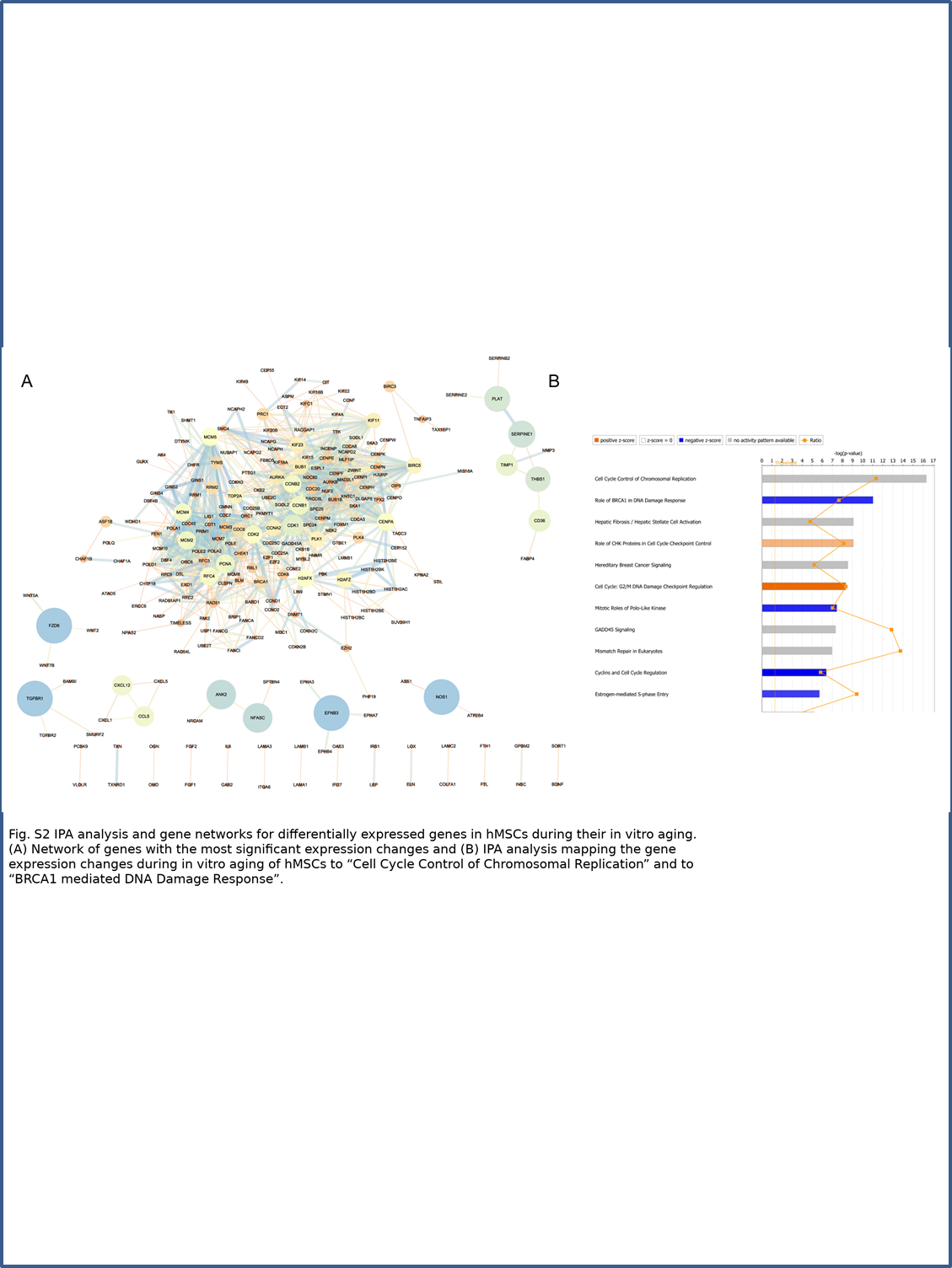

Supplement: Supplementary file 2 — Fig. S2. IPA analysis and gene network for the changed gene in in vitro aging process in hMSCs. [file FEB4-10-1238-s002.tif]

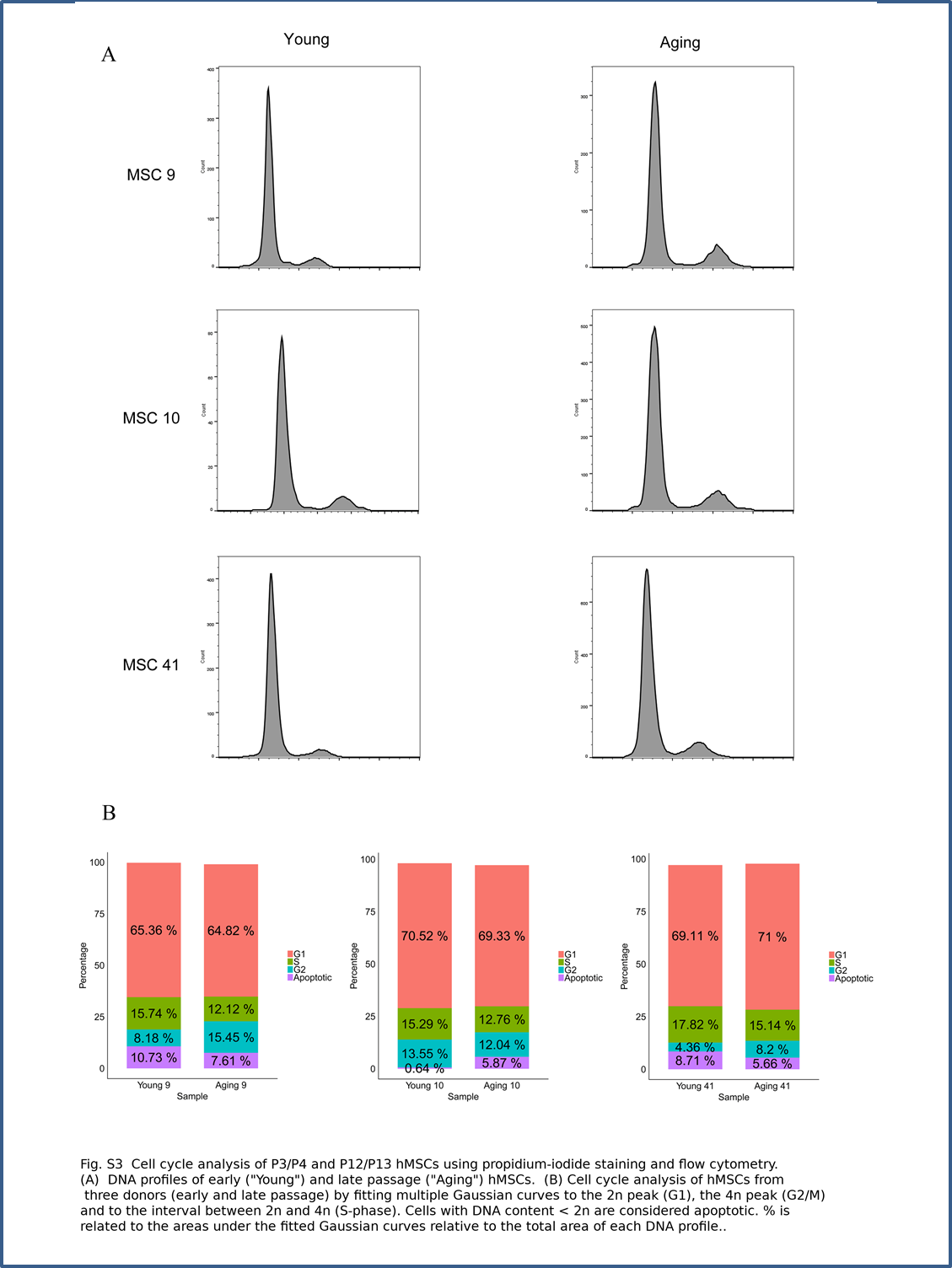

Supplement: Supplementary file 3 — Fig. S3. Cell cycle analysis of P3/P4 and P12/P13 MSCs using propidium iodide staining and flow cytometry. [file FEB4-10-1238-s003.tif]

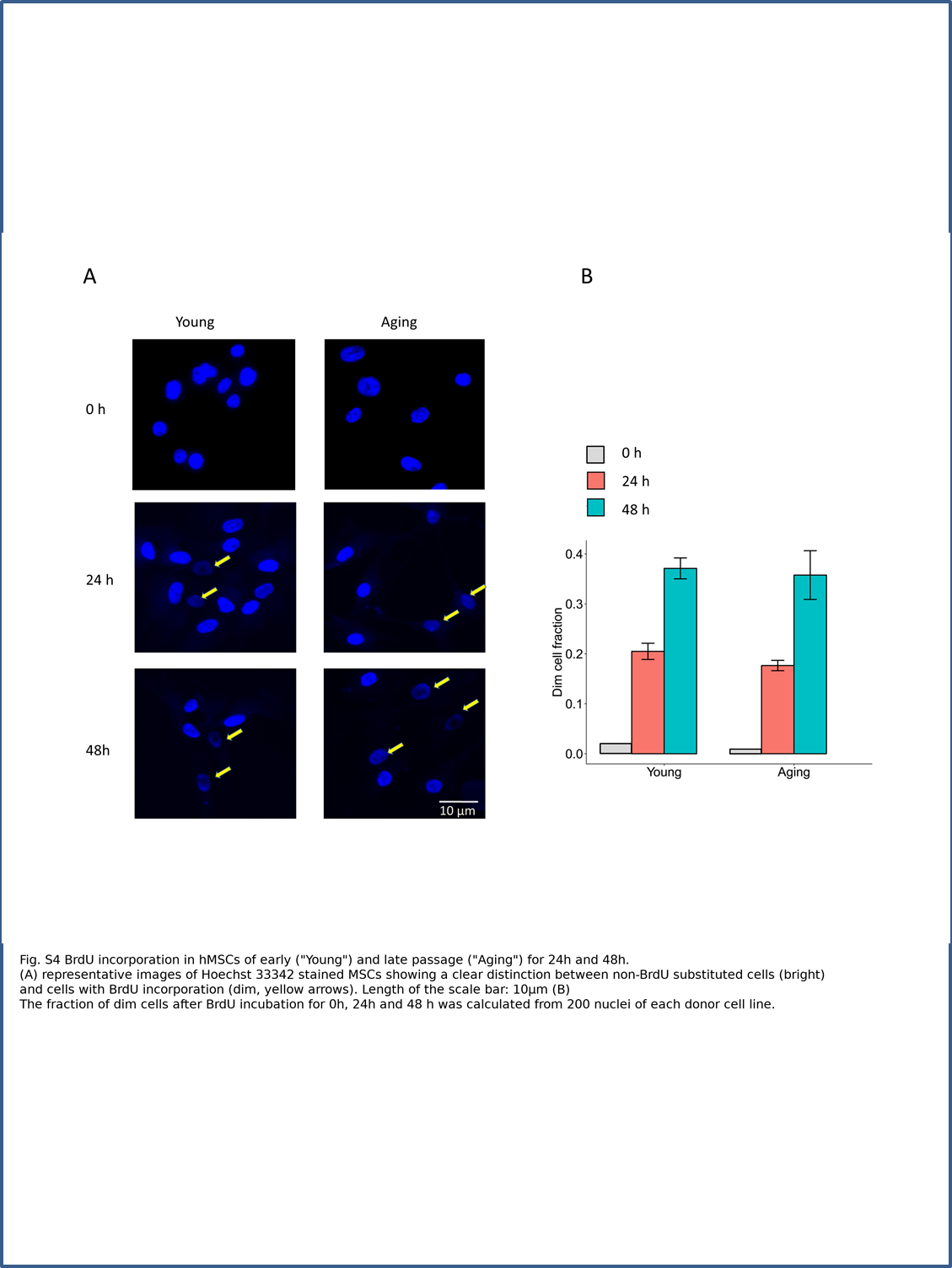

Supplement: Supplementary file 4 — Fig. S4. BrdU incorporation for 24 and 48 h. [file FEB4-10-1238-s004.tif]
