# Supplementary material for: Extended in vitro culture of primary human mesenchymal stem cells downregulates Brca1‐related genes and impairs DNA double‐strand break recognition
Source: FEBS Open Bio. 2020 Jun 9;10(7):1238–50. doi: 10.1002/2211-5463.12867 (PMC7327915; doi:10.1002/2211-5463.12867)
Supplement: Supplementary file 5 — Table S1. List and nucleotide sequence of primers used for qRT‐PCR. List and nucleotide sequence of primers used for qRT‐PCR. [file FEB4-10-1238-s005.doc]

**Table. S1 Primer sequences**

| **Gene symbol** | **Primer** | **Sequence** |
| --- | --- | --- |
| BRCA1 | Forward  Reverse | CTGCTCAGGGCTATCCTCTC  TGGCTCCCATGCTGTTCTAA |
| RAD51 | Forward  Reverse | CCACAACCCATTTCACGGTT  GCAACAGCCTCCACAGTATG |
| RAD54L | Forward  Reverse | GTAGAGCGCCACTTCTCTCT  GTGCAACCTGTCATGTGTGT |
| RAD54B | Forward  Reverse | GCACCTACACTGGCAACATT  AGAACAGCATCACCTTCCCA |
